# High Dosage Lithium Treatment Induces DNA Damage and p57^Kip2^ Decrease

**DOI:** 10.3390/ijms21031169

**Published:** 2020-02-10

**Authors:** Emanuela Stampone, Debora Bencivenga, Clementina Barone, Arianna Aulitto, Federica Verace, Fulvio Della Ragione, Adriana Borriello

**Affiliations:** Department of Precision Medicine, University of Campania “Luigi Vanvitelli”, 80100 Naples, Italy; emanuela.stampone@unicampania.it (E.S.); debora.bencivenga@unicampania.it (D.B.); clementina.barone@unicampania.it (C.B.); arianna.aulitto@unicampania.it (A.A.); federicaverace@gmail.com (F.V.)

**Keywords:** p57^Kip2^, LiCl, SH-SY5Y, oxidative stress, DNA damage, DNA damage response

## Abstract

Lithium salt is the first-line therapeutic option for bipolar disorder and has been proposed as a potential antitumoral drug. The effects of LiCl treatment were investigated in SH-SY5Y, a human neuroblastoma cell line and an in vitro model of dopaminergic neuronal differentiation. LiCl, at the dosage used in psychiatric treatment, does not affect cell proliferation, while at higher doses it delays the SH-SY5Y cell division cycle and for prolonged usage reduces cell viability. Moreover, the ion treatment affects DNA integrity as demonstrated by accumulation of p53 and γH2AX (the phosphorylated form of H2AX histone), two important markers of genome damage. p57^Kip2^, a CIP/Kip protein, is required for proper neuronal maturation and represents a main factor of response to stress including genotoxicity. We evaluated the effect of lithium on p57^Kip2^ levels. Unexpectedly, we found that lithium downregulates the level of p57^Kip2^ in a dose-dependent manner, mainly acting at the transcriptional level. A number of different approaches, mostly based on p57^Kip2^ content handling, confirmed that the CKI/Kip reduction plays a key role in the DNA damage activated by lithium and suggests the unanticipated view that p57^Kip2^ might be involved in DNA double-strand break responses. In conclusion, our study identified novel roles for p57^Kip2^ in the molecular mechanism of lithium at high concentration and, more in general, in the process of DNA repair.

## 1. Introduction

Since the last century, the therapeutic importance of lithium has been investigated, and many benefits of its use have been reported [1,2]. The main medical use of Li treatment is mental illness. Indeed, Li salt represents a well-established therapeutic option for bipolar disorder; it is also employed in schizophrenia, major depression, and in several psychiatric diseases when other treatments fail [3]. However, numerous toxic side effects were observed, only partially solved by the adjustment of the employed doses [2]. Safe therapeutic lithium blood levels range from 0.6 to 1.2 mM, while lithium toxicity can be observed when the serum concentration reaches 1.5 mM or higher [2]. 

Molecular mechanisms underlying Li action are complex and not completely clarified. This might be mostly due to the pleiotropy of Li molecular effects. So far, in vitro and in vivo studies have proposed that Li modulates several key enzymes and biochemical pathways in the nervous system, including inositol monophosphatase (IMP) [4], adenylyl cyclase [5], protein kinase C [6], phosphatidylinositol 3-kinases (PI3K)/AKT/glycogen synthase kinase 3 (GSK3), and WNT/beta-catenin pathways [7,8]. A main activity of Li is the inhibition of GSK3β [9], a serine/threonine kinase involved in many processes such as insulin response, cytoskeletal organization, and apoptosis [10]. However, none of the proposed biochemical mechanisms is sufficient to explain alone Li functions. 

Due to the fact of its relevant and well-established inhibition activity on pro-survival and pro-proliferative kinases, the use of LiCl in cancer therapy has also been exploited. Lithium treatment reduces the viability of several cancer cell lines of different origin, such as neuroblastomas and prostate and colon cancers [11,12,13], mainly through the induction of programmed cell death.

Mechanistically, it has been suggested that LiCl’s putative effects rely on the ability to interfere with several cellular processes and activities, such as the redox state, mitochondrial function, autophagy, DNA damage (DD), and the consequent cellular response (generally defined as DNA damage response or DDR) [14,15,16]. As a matter of fact, the maintenance of genome stability is a cornerstone of normal cell homeostasis, and DDR pathways ensure genome integrity [17]. DD has been associated with the pathophysiology of psychiatric disorders, like bipolar disorder [18], and with neurodegenerative pathologies such as Alzheimer’s and Parkinson’s [19]. In these syndromes, defects of DDR systems have been found to exacerbate amyloid precursor pathological action [19]. Genotoxic stress and apoptosis induction are also general mechanisms of anti-cancer drugs. There is evidence that the administration of Li salts to cultured cells promotes DD and, subsequently, the DDR initiation [15,16].

Molecularly, DD induces the activation of the DNA damage-sensing serine/threonine kinases ataxia-telangiectasia mutated (ATM) and ATM- and Rad3-related (ATR) kinases which are considered the master transducers of DNA signals, together with DNA-PKcs (DNA-dependent protein kinase, catalytic subunit). All these kinases are members of the phosphatidylinositol-3-kinase-like kinases (PIKKs) family [20] and might activate an ample network of signal transduction pathways able to maintain genome integrity. Among them, the phosphorylation, mainly due to the ATM, of the histone variant H2AX on Ser139, forming γH2AX, is required to initiate a chromatin-based signaling cascade which activates the recruitment of DDR proteins at the lesion’s region [21]. In parallel, p53 signaling may be activated to induce cell cycle arrest, apoptosis, and/or senescence effector proteins [22,23]. One well-known p53 target gene is *CDKN1A* that encodes p21^Cip1^, a cyclin-dependent kinase (CDK) inhibitor (CKI), which binds to the cyclin–CDK complexes and inhibits their activity, leading to cell cycle arrest. p21^Cip1^ belongs to the CDK interacting protein/kinase inhibitory protein (CIP/Kip) CKI family that also includes p27^Kip1^ and p57^Kip2^ (hereinafter p57) [24]. Among the three siblings, p57, the less characterized family member, has a peculiar role in allowing cell survival upon a variety of stresses [25,26,27,28]. Particularly, studies in murine and human cell lines revealed that p57 is part of the cellular stress response under conditions such as oxidative stress and UV exposure [26]. In accord, *Cdkn1c* (the mouse gene encoding p57, corresponding to *CDKN1C* in humans) ablated mice mostly die after birth, exhibiting an increased rate of cellular apoptosis and severe developmental defects, while p21^Cip1^- and p27^Kip1^-KO mice do not present important growth defects [25]. 

In addition, in cancer cell lines p57 seems to have a role in chemoresistance [29]. Particularly, in primary rectal cancer cells and in tumor models, it has been shown that doxorubicin administration induces p57 upregulation due to the activation of the ATM pathway. It is to underline that ATM-associated mechanisms are capable of activating the G1/S checkpoint thus preventing apoptosis [29,30]. In contrast, the overexpression of p57 has also been correlated in some instances to the promotion of apoptosis in cancer cells [28,29,31]. In addition, it has been reported that p57, in parallel with the ability to stabilize the actin cytoskeleton through modulation of cofilin phosphorylation, might translocate into mitochondria promoting Bax activation and the mitochondrial apoptotic cell death pathway [31]. These conflicting observations (favoring cell survival under stress conditions versus activating cell death) suggest a context-specific p57 role in cell death modulation.

In adults, *CDKN1C* transcription is restricted to some tissues including the nervous system [32]. Furthermore, p57 is highly expressed in several neuroblastoma cell lines [32]. Since in neural cells p57 plays important roles in the response to stress conditions, acting as a pivotal effector molecule of the DNA damage response [25,26,27,28,29], and Li activity has been related to DNA damage [12], we investigated the effect of Li on p57 levels/activity in neuroblastoma cells in connection with cell phenotype.

## 2. Results

### 2.1. Proliferation Rate and Viability Reduction of SH-SY5Y Cells Induced by LiCl Treatment

**Figure 1:**
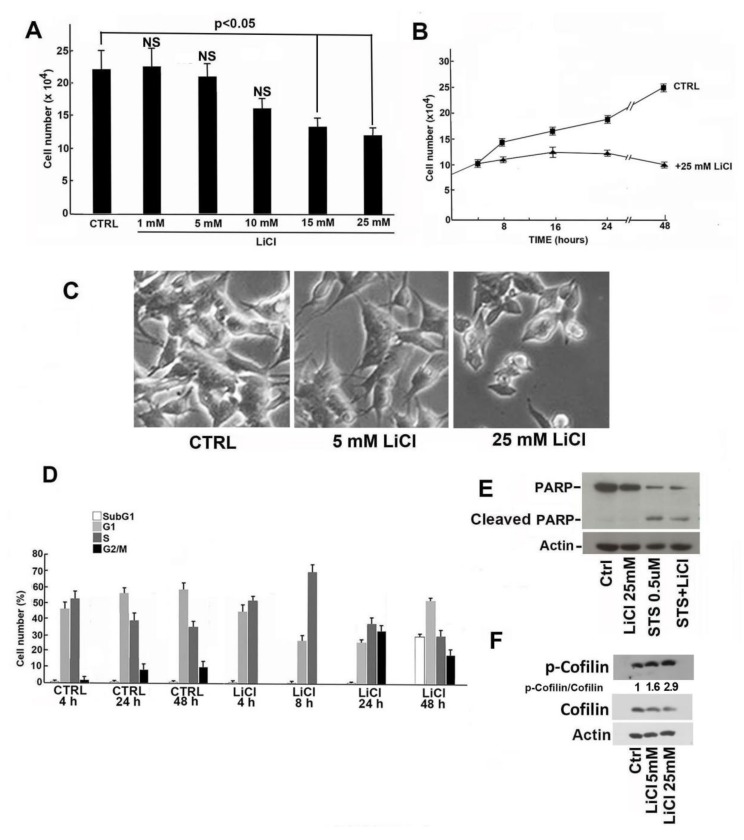
Effect of lithium on the growth and morphology of SH-SY5Y cells. (**A**) The dose-dependent effect of LiCl on the proliferation rate of SH-SY5Y cells after 24 h of incubation was evaluated by direct cell counting. The CTRL value represents the number of cells cultured with 25 mM NaCl. The data shown are the mean of three independent experiments, and the standard deviation (T bar) is reported. A *p*-value < 0.05 denotes a significant difference; NS, lack of significant difference. (**B**) Effect of 25 mM LiCl on the growth of SH-SY5Y cells at different incubation times. The data shown are the mean of three independent experiments, and the standard deviation (T bar) is reported. (**C**) Effect of two different concentrations of LiCl (5 mM and 25 mM) on the morphology of SH-SY5Y cells in comparison with cells grown in the presence of 25 mM NaCl (CTRL). The images were taken using a Leica DM IRB inverted microscope with an objective 20×/1.5. (**D**) Effect of 25 mM LiCl on the cell cycle phase distribution of SH-SY5Y cells evaluated at different incubation times. CTRL refers to the distribution of cells cultured in presence of 25 mM NaCl at the reported incubation times. (**E**) SH-SY5Y cells were treated with 25 mM NaCl (CTRL), 25 mM LiCl, and 0.5 µM staurosporine (STS) for 4 h and 25 mM LiCl for 24 h plus 0.5 µM STS added to the medium for the last 4 h of incubation. After 24 h treatment, cell extracts were prepared and analyzed by Western blotting using a monoclonal anti-PARP antibody able to detect both full-length PARP1 (116 kDa) as well as the large fragment (89 kDa) of PARP1 resulting from caspase cleavage. (**F**) The level of phospho-cofilin was analyzed in protein extracts of SH-SY5Y cells treated with 5 mM and 25 mM LiCl for 24 h in comparison with cells grown in the presence of 25 mM NaCl as CTRL. The ratio between phospho-cofilin and cofilin signal intensities was calculated using ImageJ software (see Materials and Methods, Section 5).

The effects of LiCl on cell proliferation and cell cycle distribution were investigated in the SH-SY5Y neuroblastoma cell line. Consistent with the data reported in the literature [11,12,13], LiCl induced at 24 h a dose-dependent reduction of cell proliferation (Figure 1A). A time-course experiment was then performed using 25 mM LiCl and a time interval up to 48 h. A clear growth inhibition was evident after only 8 h of incubation (Figure 1B). Lithium treatment also modified the cellular morphology (Figure 1C). Particularly, cells exposed to 25 mM LiCl for 24 h showed shorter neurite-like protrusions compared to control cells (Figure 1C).

Cell-cycle analysis of SH-SY5Y cells treated with 25 mM LiCl evidenced an early (after 4 and 8 h and up to 24 h) accumulation in the S phase with a relevant delay in the progression into G2 phase, suggesting a restraint in overpassing the S/G2 checkpoint. No apoptosis was evident after 24 h treatment (1% sub-G1 cells) while, at 48 h of incubation, apoptosis induction increased with 30% sub-G1 cells (Figure 1D). Comparatively, cell cycle analysis of SH-SY5Y control cells (i.e., treated with 25 mM NaCl) showed the absence of apoptosis and a small reduction of S phase cells. Thus, to study Li effects in its early stages and to avoid effects due to the apoptosis, 24 h treatment was chosen for the subsequent investigations. Figure 1E reports the result of an immunoblotting analysis of poly (ADP-ribose) polymerase-1 (PARP-1), an enzyme that plays a central role in DD repair and whose 90 kDa cleavage product is a recognized marker of apoptosis. As clearly shown, 24 h Li exposure did not determine PARP-1 cleavage which was conversely evident when cells were treated with 0.5 μM staurosporine (a powerful apoptosis inducer here employed as positive control), alone or in the presence of 25 mM LiCl. We also investigated the effect of Li on cofilin phosphorylation. Cofilin phosphorylation plays a major role in cytoskeleton remodeling and, accordingly, might be associated to the cell morphology changes (shown in Figure 1C). As reported in Figure 1F, Li clearly increased phospho-cofilin levels.

### 2.2. Oxidative and Genotoxic Stress Determined by LiCl Treatment

The slowing down of the cell cycle progression through the S phase might be due to the activation of the intra-S checkpoint as a mechanism to safeguard the genome stability from possible genotoxic stress due to the 25 mM Li treatment. At this concentration, Li has been reported to increase DNA damaging reactive oxygen species (ROS) [15]. 

**Figure 2:**
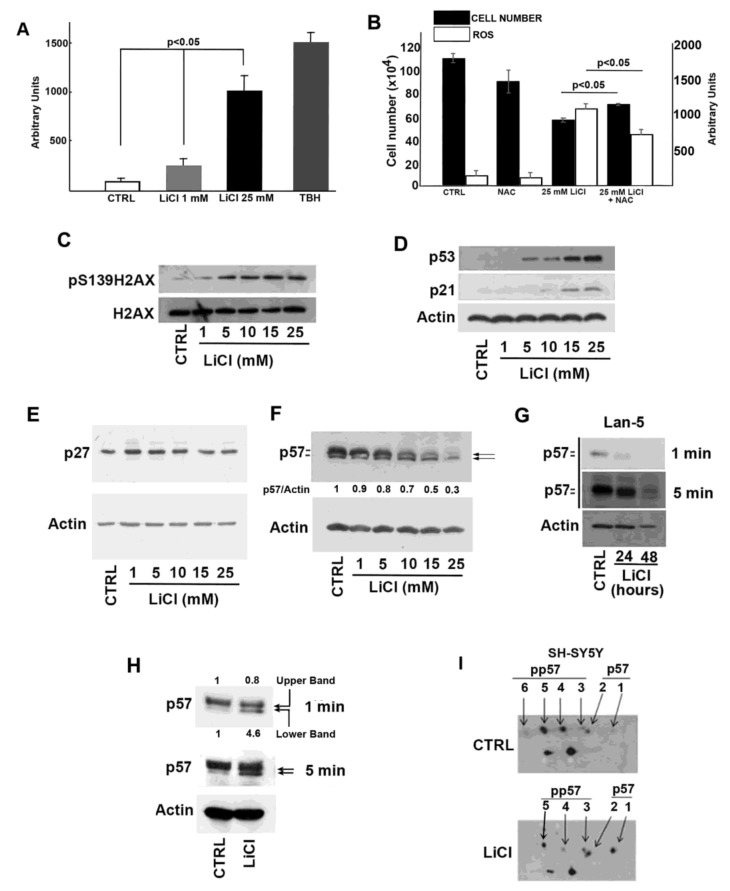
Effect of lithium on ROS production, DNA damage, and p57 cellular content. (**A**) Effect of different amounts of LiCl on intracellular ROS level in SH-SY5Y cells. Treatment with 1 mM *tert*-butyl hydroperoxide (TBH) was used as the positive control. The data shown represent the mean of three independent experiments, and the standard deviation (T bar) is reported. A *p*-value < 0.05 denotes significant difference. (**B**) SH-SY5Y cells were treated with 25 mM LiCl in the presence or absence of N-acetylcysteine (NAC). CTRL refers to cells incubated with 25 mM NaCl; NAC represents cells exposed to 25 mM NaCl plus 150 µM NAC. The growth rate was evaluated by direct cell counting after 24 h incubation. The plot also shows the intracellular ROS levels determined as in (**A**). Data are the mean of three independent experiments, and the standard deviation (T bar) is reported. A *p*-value <0.05 denotes a significant difference. (**C**) Western blotting analysis of the variation of the amount of γH2AX (pS139H2AX) and H2AX in response to the treatment of SH-SY5Y cells with different amounts of LiCl for 24 h. CTRL is represented by cells incubated with 25 mM NaCl for 24 hours. (**D**) As in panel (**C**), except that p21^Cip1^ (p21) and p53 were analyzed in the cell extracts. Equal loading of proteins was verified by determining actin content. (**E**) As in panel (**C**), except that p27^Kip1^ was analyzed. Actin content was determined to verify equal loading of proteins. (**F**) As in panel (**C**), except that p57 levels were analyzed. The ratio between p57 and actin signal intensities is reported. (**G**) Lan-5 cells were treated with 25 mM NaCl (CTRL) or with 25 mM Li for 24 and 48 h, and cell extracts were analyzed for p57 content by immunoblotting. Equal loading of proteins was verified by determining actin content. Films at two different exposure times are shown. (**H**) SH-SY5Y cells were transfected for 8 h with 1 µg p57-pcDNA3.1 expression vector. Then, 25 mM NaCl (CTRL) or 25 mM LiCl were added to the cell media for additional 24 h. Thus, equal amounts of proteins were analyzed for p57 content by immunoblotting. The arrows represent the upper and lower p57 signals. The p57-specific signals were quantified as the ratio of the upper and lower signal over actin signal, respectively. Films at two different exposure times (1 and 5 min) are shown. (**I**) SH-SY5Y cells were treated with 25 mM NaCl (CTRL) or 25 mM LiCl for 24 h. After the treatment, cell extracts were analyzed for p57 by bidimensional immunoblotting using anti-p57 mouse monoclonal Ab. The isoforms at the upper level correspond to phosphoforms with an increasing number of phosphate moieties (isoforms from 3 to 6). The lower level includes the unmodified form (signal 1) and the phosphoform with one phosphorylated residue (signal 2). Additional details are described in Materials and Methods, Section 5.

To confirm that in our cell model Li induces oxidative stress, intracellular ROS levels were measured by dichlorofluorescein (DCF) assay in cells treated with 1 and 25 mM LiCl for 24 h (Figure 2A). Cells exposed to tert-butyl hydroperoxide (TBH) for 3 h were employed as the positive control. While 24 h treatment with 1 mM LiCl had no (or scarce) effect in comparison with control cells, a statistically significant increase in the ROS level (*p* < 0.05) in 25 mM LiCl-treated cells was observed. We also evaluated whether enhanced ROS production was involved in Li-dependent cellular effects and particularly in its anti-proliferative activity. To this aim, we compared Li effects on cellular growth in the presence or absence of N-acetylcysteine (NAC), a well-known and widely used antioxidant molecule (Figure 2B). The data obtained, demonstrating only a partially protective NAC activity, correlated the Li-dependent ROS production and effect on cell growth but also suggested the occurrence of additional pathways involved in the Li mechanism of action.

To investigate the possible genotoxic activity of Li, we analyzed the levels of γH2AX (pS139H2AX), a marker of DNA double-strand breaks (DSBs), in Li-treated cells. As shown in Figure 2C, a dose-dependent increase of γH2AX immunoreactive signal was observed in Li-exposed SH-SY5Y cells. It is well-known that p53 orchestrates a variety of DNA-damage response mechanisms. Accordingly, we evaluated whether Li activates the p53/p21^Cip1^ pathway to arrest cell proliferation and to allow DNA repair. As reported in Figure 2D, a clear increase of both proteins was induced by Li. We also evaluated the effect of Li on the other Cip/Kip CDK inhibitors. While no significant variation of p27^Kip1^ cell content was evident (Figure 2E), unexpectedly, p57 levels were progressively reduced by Li addition (Figure 2F). Due to both the novelty of the last observation and the proposed key role of p57 in stress response [25,26,27,28], we focused our attention on the possible correlation between Li-induced genotoxic effect and Li-dependent p57 downregulation. 

First, we confirmed the negative Li effect on p57 cellular content in an additional neuroblastoma cell line, namely, Lan-5 cells (Figure 2G). This *N-Myc* amplified cell line was selected for comparison with SH-SY5Y cell line that does not show *N-Myc* amplification. The result obtained in Lan-5 cells suggests that the Li-dependent p57 decrease was not correlated to *N-Myc* status (Figure 2G). Usually, in immunoblotting analyses, p57 signal, as noted in Figure 2F,G, appears as a doublet where, as we recently demonstrated, the upper and slower migrating band corresponds to more phosphorylated isoform(s), while the lower band to hypo- or unphosphorylated form(s) [33]. Interestingly, beside the decrease of p57 total levels, the decline in the p57 upper band signal intensity was particularly evident (Figure 2F,G), suggesting that Li affects p57 phosphorylation. The effect was also observed, although to a minor extent, by transfecting the cells with an expression vector encoding for p57 (p57-pcDNA3.1) and adding, after 8 h, 25 mM LiCl for an additional 24 h. In this experiment, the total amount of the protein was scarcely affected, the p57 expression being controlled by the plasmid promoter (CMV). However, the analysis of the Li-incubated transfected cells showed a decrease of the upper signal associated to the accumulation of the lower band, indicating an effect on protein phosphorylation (Figure 2H). To confirm the effect of Li on p57 phosphorylation, we performed a two-dimensional electrophoresis followed by Western blotting (2D/WB) of extracts from 24 h LiCl-treated cells compared to the control, namely, 24 h NaCl-treated cells. The results showed a Li-dependent decrease of the phosphorylated specific isoforms (i.e., those focalizing at more acidic pH and with a slightly higher apparent MW), corresponding, in the control sample shown in Figure 2I, to the spots numbered from 3 to 6. Contemporaneously, Li exposure induced an intensification of the signals (spots 1 and 2) specific for the unmodified and hypophosphorylated p57 isoforms, respectively (Figure 2I).

### 2.3. Molecular Bases of Li-Dependent Effects on p57 Protein

**Figure 3:**
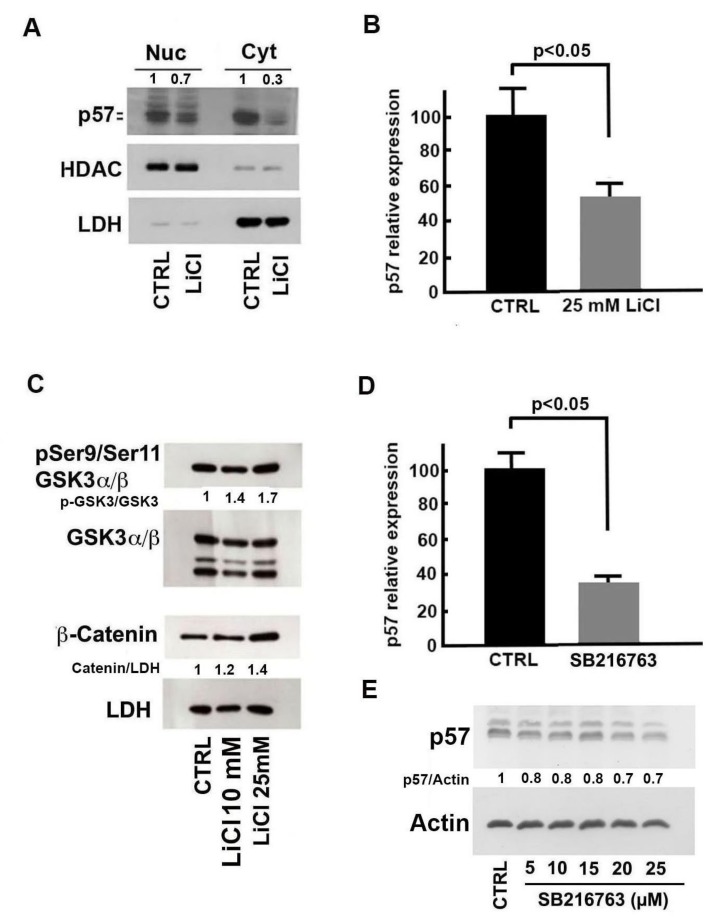
Molecular mechanisms of lithium’s effect on p57 content. (**A**) Immunoblotting analysis of nuclear and cytosolic fractions of SH-SY5Y cells treated with 25 mM NaCl (CTRL) or LiCl for 24 h. HDAC1 and LDH were analyzed to confirm the efficiency of cell compartment fractionation and equal protein loading. The ratio between p57 and the loading control signal is also reported. (**B**) SH-SY5Y cells were treated with 25 mM NaCl (CTRL) or 25 mM LiCl for 24 h, and the *CDKN1C* transcript was evaluated by quantitative RT-PCR. The data shown are the mean of three independent experiments, and the standard deviation is reported. A *p*-value < 0.05 denotes significant difference. (**C**) SH-SY5Y cells were treated with 25 mM NaCl (CTRL) and with 10 and 25 mM LiCl for 24 h. Cell extracts were analyzed for phosphoserine21-phosphoserine9/GSK3α/β (pSer21/pSer9-GSK3α/β) and β-catenin content by immunoblotting. Equal loading of proteins was verified by determining the LDH content. The ratios between pSer21/pSer9-GSK3α/β and GSK3 α/β and β-catenin and LDH band intensities are also shown. (**D**) Effect of SB216763 treatment on *CDKN1C* expression. SH-SY5Y cells were treated with 25 µM SB216763 or with the vehicle DMSO (CTRL) for 24 h. *CDKN1C* expression was evaluated by quantitative RT-PCR. The data shown are the mean of three experiments, and the standard deviation is reported. A *p*-value < 0.05 denotes a significant difference. (**E**) SH-SY5Y cells were treated with different amounts of SB216763 for 24 h. Cell extracts were analyzed for p57 content by immunoblotting. The ratio between p57 and actin signal intensities is also shown. Further details for the densitometric analysis reported in panels (**C**) and (**E**) are described in Materials and Methods, Section 5.

Since the p57 protein, same as its siblings p21^CIP1^ and p27^Kip1^, plays numerous roles in cell biology depending on subcellular localization and on specific interactors and might also have a different regulation in distinct cell compartments, the nuclear/cytosolic distribution of the protein in cells treated with 25 mM LiCl for 24 h was evaluated. As shown in Figure 3A, Li reduced p57 levels in both compartments and particularly in the cytosol. To unravel the molecular mechanisms by which Li treatment reduces p57 levels, we evaluated the effects of LiCl (25 mM at 24 h) on the transcription of *CDKN1C*. The results of a real-time RT-PCR experiment, reported in Figure 3B, show approximately a 45% downregulation of the *CDKN1C* transcript in treated cells compared to the control. Overall, our findings demonstrate that Li affects p57, both by reducing *CDKN1C* gene transcription (Figure 3B) and altering protein post-translational modifications (Figure 2I). In addition, we observed that the negative effect mostly affects the protein localized in the cytosolic compartment.

Lithium is considered a prototypical inhibitor of GSK3α/β, a multifunctional kinase involved in many pathways that regulate a variety of biological processes. The best characterized is the TKR-(or PI3K)-AKT-GSK3-WNT/β-catenin pathway. Particularly, β-catenin is a substrate of GSK3β. The GSK3β-dependent phosphorylation targets the protein for ubiquitination and subsequent proteasome degradation therefore contributing to its regulation [34]. In our model, Li treatment gives a dose-dependent increase of phosphoSer9/phosphoSer11-GSK3 (the inactive form of GSK3β) levels and a more evident β-catenin accumulation (Figure 3C). This is in agreement with data found in the literature reporting a Li-dependent increase of the inhibitory N-terminal phosphorylation of the enzyme, possibly by an autoregulation mechanism [34]. Therefore, we investigated the activity of SB216763, a selective inhibitor of GSK3, on p57, both at the mRNA and protein levels. While 25 µM SB216763 exposure for 24 h strongly reduced *CDKN1C* gene transcription (Figure 3D), dose-dependent experiments (5–25 µM) showed a modest (approximately 30%) decrease of p57 protein (Figure 3E). These findings suggest that GSK3 inhibition might explain only in part Li activity on p57 cellular content. On the other hand, it has been reported that the Wnt/β-catenin pathway downregulates *CDKN1C* expression during midbrain dopaminergic neuron development, promoting cell cycle progression of neural precursor cells [35]. Thus, it is reasonable to hypothesize that LiCl upregulates β-catenin (Figure 3C) by GSK3-dependent and/or -independent mechanisms and that this event results in p57 downregulation. 

### 2.4. Role of p57 Decrease in Li-Dependent DNA Damage Response 

**Figure 4:**
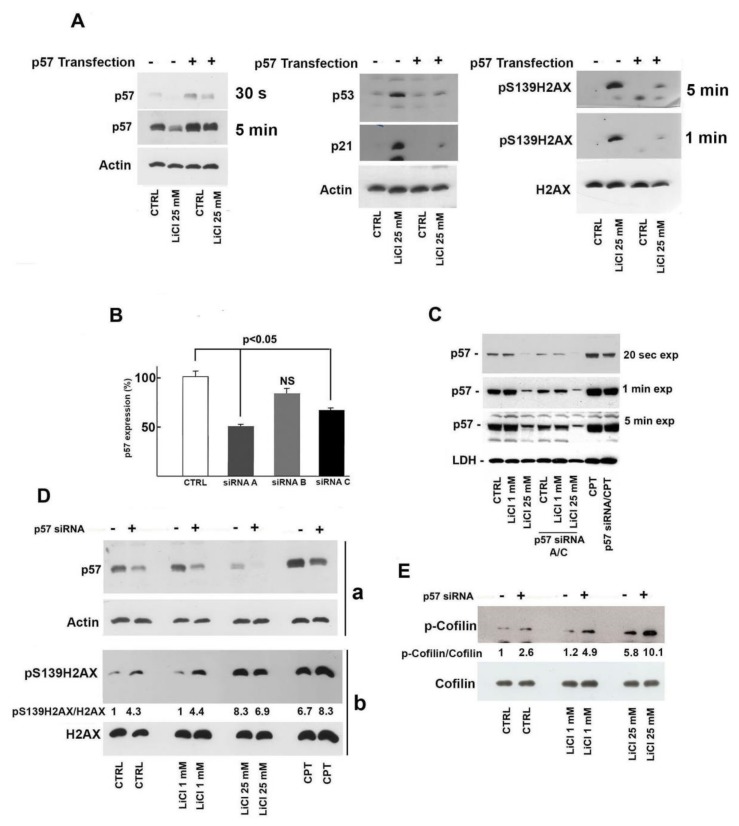
Importance of the decrease of p57 in lithium-dependent DNA damage. (**A**) SH-SY5Y cells were plated in multiple wells at equal density (50–60% of confluency). After 24 h, an equal number of cells were transfected with 300 ng of a p57-encoding pcDNA3.1 plasmid or with a pcDNA3.1 empty vector; then, 8 h after transfection, each cell population was exposed to 25 mM LiCl or to 25 mM NaCl (CTRL) for 24 h. Cells extracts were analyzed by immunoblotting for specific proteins, as reported (p57 on the left, p53 and p21 in the middle, pSer139/H2AX and total H2AX on the right). Two films at different exposure times (30 s and 5 min) for p57 and for pSer139/H2AX (1 and 5 min) are shown. (**B**) Three different siRNAs directed against *CDKN1C* (named siRNA A, B, and C) were transfected (at 100 nM concentration) in SH-SY5Y cells (see Materials and Methods, Section 5). Forty-eight hours after transfection, cells were harvested, and total RNA was prepared. *CDKN1C* expression was evaluated by quantitative RT-PCR. The data shown are the mean of three experiments, and the standard deviation (T bar) is reported. (**C**) SH-SY5Y cells were first transfected with a mixture of anti-p57 siRNA A and siRNA C (siRNA A/C) for 48 h and thereafter exposed to 1 mM and 25 mM LiCl for 24 h; a specific control included cells incubated for 24 h with 25 mM NaCl after 48 h of transfection with a scramble siRNA. The transfected cells were also compared to SH-SY5Y cells treated with the two different Li concentrations (1 mM and 25 mM) for 24 h. In addition, SH-SY5Y cells were treated with 2 µM CPT (camptothecin) for 5 h after transfection (or not) with siRNAs. Then, equal amounts of proteins were analyzed for p57 content by immunoblotting. LDH was used as equal loading control. Three different exposure times are shown to highlight signal differences. (**D**) SH-SY5Y cells were treated with NaCl (CTRL) or with LiCl at two different concentrations in the presence or absence of the siRNA mixture. Moreover, cells were treated with 2 µM CPT. After 24 h, cell extracts were prepared and analyzed. In (**a**), p57 and actin levels were investigated while, in (**b**), γH2AX (pS139H2AX) and H2AX were studied. The ratios between p57/actin and γH2AX/H2AX were also reported. (**E**) The samples in (**D**) were analyzed for phosphoserine3-cofilin and cofilin content. The ratio between phosphocofilin and cofilin signal intensities is also shown. Details of the densitometry analysis are in Materials and Methods, Section 5.

As previously shown, Li determined a progressive dose-dependent increase of γH2AX and p53 that is associated to p57 reduction. Since p57 has been demonstrated to act as a positive effector in survival response under stress conditions, we hypothesized that p57 downregulation might play a role in Li-dependent DNA damage in SH-SY5Y cells. To confirm this hypothesis, we decided to handle p57 level (both increase and decrease) and investigate possible change of Li genotoxicity. Thus, SH-SY5Y cells were transfected with p57-pcDNA3.1 and then treated with 25 mM Li. The data reported in the left panel of Figure 4A confirmed the transfection efficiency. More importantly, as shown in Figure 4A (center and right panels), p57 forced expression clearly restrained the Li-dependent build-up of γH2AX, p53, and p21. 

Conversely, p57 cellular content was negatively regulated by a Li-independent approach, i.e., small interfering RNA (siRNA) treatment and, thereafter, the effect on DNA integrity of the gene silencing, in combination (or not) with Li treatment, was evaluated. The efficiency of different p57 siRNAs (here defined as siRNA-A, -B, and -C) was preliminarily investigated by RT-PCR. Two out of three siRNAs tested (i.e., A and C) reduced p57 transcript levels by roughly 50% compared to control cells treated with scramble siRNA (Figure 4B). Thus, a siRNA (A plus C) mixture was used to downregulate p57 protein levels. As showed in Figure 4C, siRNA’s addition markedly reduced p57 cellular content. This reduction was similar in the control cells and in cells treated with 1 mM Li, while in cells treated with 25 mM Li, siRNA transfection further enhanced the negative Li activity on p57. Different time exposure of immunoblot are shown in Figure 4C, allowing for a clear detection of the siRNA treatment’s effect. In the experiment shown in Figure 4C, SH-SY5Y cells were also treated with 2 µM camptothecin (CPT) for 5 h as a positive control. Camptothecin is an antineoplastic agent that blocks the activity of DNA topoisomerase thus causing high levels of DSBs [36]. Interestingly, CPT remarkably increased p57 cellular content, and the treatment with siRNA was still capable of reducing the enhancement of p57 levels.

Subsequently, we investigated the entity of DSBs in siRNA-treated cells by evaluating γH2AX levels. As reported in Figure 4D, p57 downregulation increased the signal of the DNA alteration marker. The effect was similarly observed in control cells and in cells treated with low Li concentration (1 mM). The ratio between phosphorylated and total histone was comparable, strongly suggesting that the p57 protein level should play a role in DSB. At 25 mM Li, p57 siRNA treatment did not increase the effect of the ion, probably since Li by itself strongly reduces p57. On the other hand, only a partial p57 silencing increased CPT activity in inducing DSB, confirming the general view that the expression of p57 promoted by genotoxic agents might be important for DNA integrity under stress conditions. We also evaluated cofilin phosphorylation, since, as previously reported in Figure 1F, the post-synthetic modification increased after Li treatment. Figure 4E shows that siRNA treatment (and p57 decrease) enhances phospho-cofilin levels, exerting an additive effect on Li treatment. The activity of siRNA exposure was observed in all the conditions analyzed. These data support the hypothesis that the downregulation of p57 might play a role in augmenting Li-dependent effects on actin dynamics in SH-SY5Y cells, an event that might be associated to cell motility, induction of apoptosis and also to DNA damage response.

## 3. Discussion

Here we report the unprecedented observation that Li downregulates, in neuroblastoma cells, the p57 level by reducing *CDKN1C* transcription and also affects its post-translational modifications. p57 is a multifunctional protein involved in the control of growth, differentiation, gene expression, cytoskeletal organization, and apoptosis [24,33,37]. Recent pieces of evidence suggest that p57 plays a vital role in the maintenance of cellular homeostasis under stress conditions including those correlated with genotoxic response and activation of programmed cell death [27]. Several of these activities are executed independently of its CDK inhibitory functions and might be related to the plasticity of the protein [38]. Thus, Li’s effect on p57 content might be relevant in the complex molecular activity of the ion.

In our cell model, Li induced DNA damage, specifically DSBs, as directly demonstrated by the enhanced phosphorylation of H2AX histone. In addition, Li reduced cell cycle progression to allow DNA damage repair (possibly through p53 and p21^Cip1^ accumulation), while over prolonged time, it may induce apoptosis (Figure 1D). Contemporaneously, Li reduced p57 cellular content. So far, p57 has never been associated to Li’s effects on neural cells, despite the reported relevance of p57 in the nervous system and, particularly, in midbrain dopamine cells [35]. The mechanisms by which Li affected p57 cellular content involved downregulation of *CDKN1C* expression. Moreover, a clear alteration of p57^.^ phosphorylation occurred in Li-treated cells, suggesting that post-translational modifications might also play a role in the downregulation of the protein or in modifying its activity.

With GSK3β being one of the best known LiCl target, we hypothesized that the inhibition of the kinase activity might be responsible for the transcriptional reduction of *CDKN1C* expression and/or changes in the protein phosphorylation. GSK3β is considered a master regulator gene of neural progenitor cells [39], and in several cell populations it regulates numerous pathways including those activated by Wnt receptors through the modulation of β-catenin turnover [40]. β-Catenin is a dual function protein, playing roles in cell adhesion and gene transcription, and its GSK3β-mediated phosphorylation induces the destabilization of the protein [41]. It has been reported that Wnt/β-catenin pathway reduces *CDKN1C* expression during midbrain dopaminergic neuron development, promoting cell cycle progression of neural precursor cells [35]. Thus, since an increase of β-catenin protein levels following treatment with LiCl were detected, we hypothesized that the downregulation of p57 by Li might be due, at least in part, to the fact of GSK3β inhibition and the subsequent β-catenin activation. To evaluate this hypothesis, we treated SH-SY5Y cells with SB216763, a specific GSK3 inhibitor and observed a clear reduction of the p57 transcript. On the other hand, the analysis of p57 protein levels after SB216763 treatment did not show a remarkable decrease of the protein, suggesting that other mechanisms, probably not GSK3β related, are involved in Li-dependent p57 downregulation. An intriguing and alternative possibility relies on the reported capability of ROS to activate, independently of GSK3β, the Wnt/β-catenin pathway [42] which, in turn, might affect *CDKN1C* expression. However, further investigations are needed to shed light on this possible mechanism.

A p57 increase has been suggested as a mechanism of defense against stress conditions [25,26,27,28]. Therefore, we hypothesized that the Li-dependent decrease of p57 might be involved in or might enhance Li-induced DNA damage. To evaluate whether p57 downregulation plays a role in Li-associated accumulation of DSBs, we increased p57 intracellular levels by transfecting the p57 expression vector. As reported in Figure 4A, p57 overexpression clearly reduced the upregulation of the major markers of DNA damage induced by Li treatment. The finding represents compelling evidence that the Li-dependent p57’s decrease being involved in the DNA damage induced by the ion. 

To further strengthen this hypothesis, we performed *CDKN1C* knockdown by specific siRNA (Figure 4B,C) and analyzed the changes of the phosphorylation state of H2AX and cofilin, two important events associated to DSB and actin cytoskeletal remodeling, respectively. The major result of these experiments was the observation that, in control cells or in cells treated with 1 mM Li (a concentration which neither affects p57 levels nor causes H2AX and cofilin phosphorylation), the reduction of p57 by siRNA increased the post-translational modifications of both proteins (Figure 4D,E). As matter of fact, the importance of p57 in DSB response was also confirmed by the observation that the treatment with CPT, a well-known inducer of DSBs, strongly upregulated p57 (Figure 4C) and that *CDKN1C* silencing in CPT-treated cells further increased γH2AX levels (Figure 4D).

It must be stressed that the CPT mechanism of action (i.e., inhibition of topoisomerase) appeared to be distinct from that of Li. The CPT-dependent increase in p57 hints that p57 upregulation follows DNA damage and is involved in the cellular response to DSBs. The precise role of p57 in the DNA damage response appears to be, although evidenced in this and other studies, still unclear and warrants further investigations.

In conclusion, our study furnishes novel information on Li molecular effects by demonstrating that the ion decreases the level of p57. This unreported activity appears to augment the DNA damaging activity of the Li. Moreover, and probably more importantly, our findings suggest the unexpected conclusion that p57 cellular levels play a general role in the modulation of DSBs. Given the relevance of DSBs and DSB responses in a plethora of biological and clinical conditions, including malignant transformation and chemotherapy efficacy, further investigations are warranted to clarify the precise function of p57 in DNA stability. Finally, the unanticipated interplay among p57 content, p53 level, and H2AX phosphorylation needs to be mechanistically investigated.

## 4. Critical Assessment on Lithium-Employed Concentrations and Dosages

For more than 60 years, lithium has represented a preferred treatment for bipolar and mood disorders. However, lithium is a drug with a narrow therapeutic index, namely, a drug with little difference between the therapeutic and toxic doses. As a matter of fact, lithium salt is still the primary therapy for bipolar disorder but, at the same time, the nervous system is the primary target of lithium toxicity. The dosage generally employed corresponds to a serum concentration of about 0.8–1.2 mM [43]. This value is clearly different and lower compared to the concentrations employed in this study as well as in a large number of reports on lithium cellular effects reported in literature [43]. Thus, this report might be mainly considered as an investigation evaluating the mechanisms of lithium effects at toxic concentrations. However, some additional aspects should be taken into consideration. First, the reported experiments demonstrate that lithium, at 5–10 mM, is able, after 24 h treatment, to induce a clear γH2AX increase and p57 decrease (Figure 2). Nevertheless, there are no data regarding lithium effects (during a chronic therapeutic treatment) on the cellular content of p57, a molecule that plays important roles in the physiology/pathology of neuronal cells. Thus, a potential effect on p57 at prolonged lithium therapeutic dosage might not be ruled out. Second, our study employs concentrations of the drug that have been frequently used in investigations evaluating lithium as an anti-proliferative agent in the treatment of numerous cancers, including glioma [44], colorectal cancer [45], medulloblastoma [46], hepatocellular carcinoma [47], and other tumors [48]. Although the dosages employed in these studies were clearly toxic, the development of delivery strategies specifically targeting cancer cells is conceivable. Finally, this investigation points to the downregulation of p57 (obtainable through molecular approaches independent of Li treatment) as a mechanism for enhancing the effect of antitumoral DNA-damaging drugs (like camptothecin, topotecan, irinotecan, doxorubicin, etc.). Under this view, our findings might be useful in the development of novel anticancer approaches based on the identification of new targets for potentiating the activity of already clinically employed drugs or for reducing their doses and negative side effects.

## 5. Materials and Methods

### 5.1. Cell Culture and Treatments

Human neuroblastoma SH-SY5Y and LAN-5 cell lines (ATCC, Manassas, VA, USA) were cultured in RPMI (Gibco, Thermo Fisher Scientific, Waltham, MA, USA), supplemented with 10% fetal bovine serum (FBS, Invitrogen, Thermo Fisher Scientific), 100 U/mL benzylpenicillin, 100 mg/L streptomycin (Gibco), and cultured in a humidified incubator with 5% CO_2_ at 37 °C. To investigate Li effects, LiCl (Sigma, St. Louis, MO, USA) was added to the culture medium for increasing times and doses; NaCl (Sigma) was used in the control treatment. The effect on the growth rate was evaluated by direct cell counting after 24 h incubation at different concentrations of LiCl (from 1 mM to 25 mM) and after different durations of incubation with 25mM LiCl. Twenty-five mM NaCl was used as control in all experiments where LiCl effects were exploited. The data are shown as the mean of three independent experiments, and the standard deviation (T bar) is reported. The GSK3α/β inhibitor SB216763 (Sigma) was solubilized in DMSO (Sigma) and used at increasing concentrations in the culture medium from 5 µM to 25 µM. Control cells were in this case exposed to the maximum DMSO concentration reached. Two μM CPT (Sigma) treatment was used as positive control of DSB induction. All the other reagents were of the highest purity grade commercially available.

### 5.2. Analysis of Cell Cycle Distribution 

The cell layers under investigation were harvested and centrifuged at 800× *g* for 5 min. The cell pellets were washed in phosphate buffered saline (PBS) and then permeabilized with a hypotonic solution containing 10 mM sodium citrate, 2 mM propidium iodide, 20 mM DNase-free RNase (Sigma), and 1% NP-40. The cells were incubated at room temperature for 30 min in the dark and then analyzed using FACScalibur (Becton Dickinson, CA, USA). Cell cycle distribution was calculated from 30.000-40.000 events with ModFit LTTM software (Becton Dickinson) [49].

### 5.3. Real-Time RT-PCR Analyses

Total RNA from SH-SY5Y was extracted using TRIzol reagent (Invitrogen) [50,51]. Two micrograms of total RNA were reverse transcribed into complementary DNA. The p57 mRNA levels were quantified by real-time RT-PCR (RT-qPCR) using QuantiTect Primer Assays, cod. Hs_CDKN1C_1_SG cat. Num. QT00018018 (QIAGEN, Hilden, Germany) and normalized with *18S* (QIAGEN) expression used as an internal control. Sequences of the used Primers are: *18S* Fw, 5′-AAACGGCTACCACATCCAAG-3′; Rev, 5′-CCTCCAATGGATCCTCGTTA-3′. The amplification was performed in an ABI PRISM® 7900 Sequence Detection System (Applied Biosystems, Monza, Italy) according to protocols supplied. Relative quantification was executed by ∆∆Ct methods [52] and obtained from, at least, three independent experiments. Data with a significance of *p* < 0.05 were accepted.

### 5.4. Cell Extracts Preparation and Mono- and Two-Dimensional Western Blotting Analyses

For whole cell extract preparation, cells were lysed in ice-cold RIPA buffer containing 50 mmol/L Tris-HCl, pH 8.0, 150 mmol/L NaCl, 1% NP-40, 1% sodium deoxycholate, 0.1% sodium dodecylsulfate, 0.1 mmol/L dithiothreitol, 0.05 mmol/L phenylmethyl-sulfonylfluoride, 0.002 mg/mL aprotinin, 0.002 mg/mL leupeptin, 1 mmol/L sodium orthovanadate, 40 mM sodium fluoride, and 1 mM sodium pyrophosphate decahydrate. Nuclear and cytosolic fractionation was performed as follows. Briefly, harvested cells were washed with ice-cold PBS, resuspended in ice-cold hypotonic buffer (10 mM HEPES, pH 7.9, 1.5 mM MgCl_2_ and 10 mM KCl enriched with protease and phosphatase inhibitors) and incubated on ice for twenty minutes, allowing them to swell. Then, IGEPAL CA-630 solution (Sigma) was added to 0.6% final concentration and, after 15 s incubation, cells were centrifuged at 10,000× *g* for 2 min. The supernatants, containing the cytosolic fraction, were transferred into new tubes. The pellet nuclei were then resuspended in RIPA buffer and incubated for 30 minutes on ice. After 15 min of centrifugation at 16,000× *g*, the supernatants (the nuclear extracts) were recovered in fresh tubes. The BioRad assay reagent (Bio-Rad, Hercules, CA, USA) was used to quantify the protein concentration. The nuclear and cytoplasmic extracts obtained were tested for cross-contamination through immunoblotting for HDAC1 and LDHα, respectively. 

Mono-dimensional SDS-PAGE (10–15% different acrylamide concentrations) was used to load and separate proteins. Upon electrophoresis, proteins were transferred onto nitrocellulose (Millipore, Burlington, MA, USA) and the membranes incubated overnight with appropriate primary antibodies. Then, HRP-conjugated secondary antibodies (Santa Cruz Biotechnology, Inc, Heidelberg, Germany) were applied to bind to and visualize primary antibodies [49]. Bidimensional analysis was performed as reported previously [53]. The following primary antibodies were employed: anti-p57 (mouse monoclonal), anti-actin (rabbit polyclonal), anti-p53 (mouse monoclonal), anti-phosphoSer3 cofilin (mouse monoclonal) and anti-cofilin (rabbit polyclonal), anti-γH2AX (mouse monoclonal), anti-PARP1 (mouse monoclonal) and anti-GSK3α/β (mouse monoclonal), anti-HDAC1 (rabbit polyclonal), all purchased from Santa Cruz Biotechnology; anti-p21^Cip1^ (mouse monoclonal) and anti phospho-GSK-3α/β (Ser21/9) (rabbit monoclonal) from Cell Signaling Technology (Leiden, The Netherlands); anti-p27^Kip1^ (mouse monoclonal), purchased from BD Transduction Laboratories (Franklin Lakes, NJ, USA). ImageJ software was used for evaluating the intensity of immunoblotting signals. The intensity of actin or other proteins was used as the control of the equal loading of the protein amount, whereas for the analysis of the phosphorylation’s variation, the intensity of the corresponding total protein signal was used as the control and to establish the changes in phosphorylation degree. The variations of a specific protein were calculated as the ratio of the intensity of the signal of interest and the intensity of the loading control. Accordingly, the fold changes of the investigated protein in the different experimental conditions were calculated dividing each ratio with the ratio of the control (considered as 1). Therefore, the value for the control was 1 and samples with a positive variation reported a number greater than 1, whereas samples with a negative variation reported a number less than 1.

### 5.5. Plasmid Transfection and Gene Silencing Using Small Interfering RNA (siRNA)

The coding sequence of p57 was cloned in pcDNA3.1 plasmid. The SH-SY5Y cells, at 60–70% confluence, were transfected with the plasmid using Lipofectamine3000 (Invitrogen) according to the manufacturer’s instructions. Control cells were transfected with the empty plasmid. After 8 h of transfection, cells were added with 25 mM LiCl for 24 h or with 25mM NaCl as CTRL. Thereafter, SH-SY5Y cells were collected and processed for subsequent analyses. The p57 siRNA and scramble siRNA were provided by Origene (OriGene Technologies GmbH, Herford, Germany). Cells at 60% confluence were transfected with 100 nmol/L siRNA using Lipofectamine3000 (Invitrogen) according to the manufacturer’s instructions. After 48 h of transfection, LiCl, at 1mM and 25mM concentrations, was added (or not) to media for further 24 h. Thereafter, cells were processed for subsequent analyses. The *CDKN1C* mRNA quantification and p57 immunoblotting were used to verify the efficiency of *CDKN1C* silencing.

### 5.6. Evaluation of Intracellular ROS Level and DNA Damage Analysis

The ROS quantification was performed using 2′,7′-dichlorofluorescein diacetate (DCF-DA, Sigma) as previous reported by Borriello et al. [49]. In these experiments, the treatment with 1 mM *tert*-butyl hydroperoxide (TBH) was used as the positive control [49]. To evaluate the effect of Li-mediated oxidative stress on the proliferation rate, SH-SY5Y cells were seeded the day before in 6 well culture plates at low density and treated for 24 h with 25 mM LiCl in combination or not with 150 μM NAC (Sigma). The NAC was added 2 h before treating SH-SY5Y cells with LiCl for 24 h. Then, the cells were counted and the average of the three experimental replicates was calculated. For DSB evaluation, Western blotting of γH2AX levels was performed. Five hour incubation with 2 µM CPT was used as the positive control for the DSB. 

### 5.7. Statistical Analysis

Experimental data are presented as mean values ± standard deviation. Comparison between groups of determination was performed using Sample *t*-test. A value of *p* < 0.05 was considered statistically significant.
